# Step by Step Derivation of the Optimum Multistage Compression Ratio and an Application Case

**DOI:** 10.3390/e22060678

**Published:** 2020-06-18

**Authors:** Ignacio López-Paniagua, Javier Rodríguez-Martín, Susana Sánchez-Orgaz, Juan José Roncal-Casano

**Affiliations:** ETSI Industriales, Universidad Politécnica de Madrid (UPM), José Gutiérrez Abascal 2, 28006 Madrid, Spain; javier.rodriguez.martin@upm.es (J.R.-M.); susana.sanchez.orgaz@upm.es (S.S.-O.); jj.roncal@alumnos.upm.es (J.J.R.-C.)

**Keywords:** compressors, pressure, multistage, non-equal efficiency, multistage compression, optimisation, optimum compression ratio, Lagrange multipliers, reciprocating compressors

## Abstract

The optimum pressure ratio for the stages of a multistage compression process is calculated with a well known formula that assigns an equal ratio for all stages, based on the hypotheses that all isentropic efficiencies are also equal. Although the derivation of this formula for two stages is relatively easy to find, it is more difficult to find for any number of stages, and the examples that are found in the literature employ complex mathematical methods. The case when the stages have different isentropic efficiencies is only treated numerically. Here, a step by step derivation of the general formula and of the formula for different stage efficiencies are carried out using Lagrange multipliers. A main objective has been to maintain the engineering considerations explicitly, so that the hypotheses and reasoning are clear throughout, and will enable the readers to generalise or adapt the methodology to specific problems. As the actual design of multistage compression processes frequently meet engineering restrictions, a practical example has been developed where the previous formulae have been applied to the design of a multistage compression plant with reciprocating compressors. Special attention has been put into engineering considerations.

## 1. Introduction

For minimum power consumption in industrial applications, gases should ideally be cooled at the same time they are being compressed [1], maintaining their initial temperature as constant during the whole process [2]. The increase in power consumption caused by compressing a gas that is progressively getting hotter, with large mass flows and long operating hours can be economically unsustainable [3,4,5].

However, this is not possible, so large compressions from a given $P_{in}$ to a much higher $P_{out}$, are split in smaller stages: one stage compresses the gas at a certain intermediate pressure; it is then cooled and sent to the inlet of the next, and the process is repeated until $P_{out}$. Although it is not ideal, the savings of multistage compression can be huge [6], depending on the number of stages into which the total compression is split, and how the total pressure ratio, $r_{t}=P_{out}/P_{in}$, is shared between them. The former might be given by economics; the latter is a technical issue and will be assessed here.

If all of the compressors of a *n*-stage compression have the same isentropic efficiency, η, there exists a well known formula in engineering [7] that defines the optimal compression ratio for each stage:

(1) $$r=r_{t}^{1/n}$$

This Formula (1) is generally used when designing multistage compression plants, by assuming an equal isentropic efficiency for all stages. Normally, a conservative value for efficiency is taken for preliminary design. The derivation for the case of n=2 can be found in a number of sources [8,9], but it is hard to find for any number of stages. It can be obtained with complex optimisation techniques, like in [10]. Although an extremely interesting example of the power of the method, complex techniques frequently obscure the engineering interpretation.

However, in engineering, it is seldom the case in which all conditions apply to allow using expressions, like the previous with all propriety. The interest is frequently on the basis, reasonings, and formulations, leading to the expression than on the expression itself, because they enhance the understanding of a problem and give inspiration for finding solutions. General, often simplified, methods can prove an extremely valuable tool for preliminary engineering, assuming hypotheses, estimating solutions, and guess values in numerical simulations [11] or designing methodologies.

Although elaborate formulations may reach optimal solutions that are capable of reflecting problem specificalities and details [12,13], generalisation might result in being difficult. A general point of view has been adopted here, while assuming polytropic compression, constant isentropic efficiency across the range of operation of the *n* compressors, and no head drop between stages.

This paper will develop the derivation of (1) step by step. Section 2 will define the problem, concepts, and notation. Section 3 will develop the optimisation using Lagrange multipliers; a summary of the method can be found in [14].

In the engineering of multistage compression plants, after the pressure ratios have been set according to (1), the actual operating conditions of each stage are determined according to manufacturer specifications. Logically, this will show that each stage will be operating with a different isentropic efficiency. Sometimes engineering constraints do not allow for reaching the intended compression ratio at a certain stage [15].

The optimum compression ratio that should be set in the case of different stage isentropic efficiencies is usually not calculated in practice. A numerical calculation has been developed in [16]. However, this work will solve the problem analytically in Section 4.1. This will show that the standard compression ratio for equal stage isentropic efficiencies must be scaled for each particular stage, depending on how much its efficiency deviates from the geometric mean. The optimum total specific work will also be analytically derived in Section 4.2. It will be discussed how different stage isentropic efficiencies tend to increase compression work, even using the stage optimum compression ratio.

Finally, the example of Section 5 illustrates how design requirements and compressor specifications combine when designing compression plants. There exists a wide variety of compressor technologies [17,18] that are generally selected, depending on the application. However, the principle of operation of reciprocating compressors, based on a cylinder and a piston, results in being intuitive [19], so a reciprocating compressor will be considered. Section 5.2 will illustrate how the flow and compression requirements might not be met with single stage compression; Section 5.3 will assess multistage dimensioning.

## 2. Problem Overview

A gas is going to be compressed in several stages from an initial pressure $P_{1}$ to an outlet pressure $P_{n+1}$, while using intermediate cooling between stages and aftercooling. The problem consists in calculating:

1. optimum pressure ratio for each stage;
2. optimum compression specific work; and,
3. amount of cooling for the optimum case.

**Figure 1:**
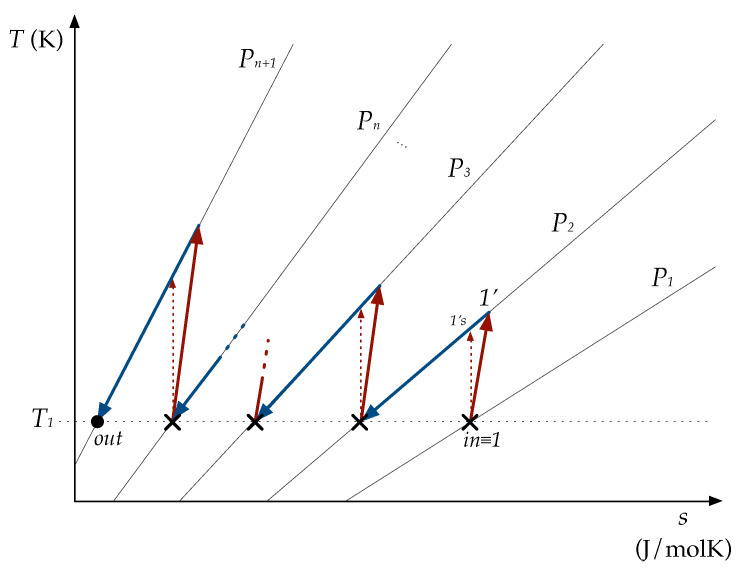
Schematic diagram of the process on a T−s chart. The inlet is $T_{1},P_{1}$, indicated by ‘*in*’; the outlet is ‘*out*’. In between there are *n* compression stages—in red—and the corresponding aftercooling stages, in blue. Thus, after a compression-aftercooling sequence (indicated by ‘x’), the gas is at $T_{1}$, but at a higher pressure each time.

The inlet and outlet conditions of the whole compression are: $\left(P_{1},T_{1}\right)$ y $\left(P_{n+1},T_{1}\right)$. The outlet temperature is kept at $T_{1}$ with the cooling. In Figure 1, the process is schematically shown on a T-s diagram.

Between $P_{1}$ and $P_{n+1}$, there are n−1 intermediate pressure levels and $P_{i},\;i=2,\ldots ,n$. The problem consists in calculating each of these values so that the full compression require minimum work.

### 2.1. Intermediate Pressure and Pressure Ratios

The term pressure ratio, *r*, will indicate the ratio between its inlet and outlet pressures. For example, the pressure ratio of a given stage between intermediate pressures *i* and i+1 is: $r_{i,i+1}=\frac{P_{i+1}}{P_{i}}$.

It can be observed that the following expression holds:

(2) $$\prod_{i=1}^{n}r_{i,i+1}=\frac{P_{2}}{P_{1}}\frac{P_{3}}{P_{2}}\ldots \frac{P_{n+1}}{P_{n}}=\frac{P_{n+1}}{P_{1}}=r_{t}$$

That is, the product of the pressure ratios of all stages gives the total pressure ratio.

Logically, this will hold whether compression work is optimised or not. That is, the intermediate pressure ratios, $r_{i,i+1}$, must satisfy this relation, even if they do not correspond to the minimum work, they cannot have any value freely.

At the time of formulating the minimum compression work, this will appear as a boundary condition.

### 2.2. Specific Work

The term specific work is the necessary work to compress a unit of gas (1 kg, one mole) a given pressure ratio. In this case, two types of specific work will be considered: the specific work between any two pressure levels on one side and the total specific work, from initial $P_{1}$ to the final $P_{n+1}$. Logically, this last is the one to minimise.

The total specific work assuming reversible compression will be indicated by $w^{R}$. The specific work to compress the gas between two consecutive pressure levels, $P_{i},P_{i+1}$ with a reversible compressor will be indicated by $w_{i,i+1}^{R}$. The total specific work is the sum of the specific works of all intermediate stages:

(3) $$w^{R}=\sum_{i=1}^{n}w_{i,i+1}^{R}$$

The following Equation [8] can be used to formulate the specific work of any given compression stage:

(4) $$w_{i,i+1}=-\int_{i}^{i+1}vdP-\Delta e_{k}-\Delta e_{p}-\psi$$

where i,i+1 indicate the inlet and outlet states.The purpose of a compressor is to increase the pressure of a gas; thus, any other effect is negligible: increments of kinetic and potential energy, $\Delta e_{k}$ and $\Delta e_{p}$, can be taken as zero. In a reversible compression, irreversibility is zero, thus ψ=0. Afterwards:

(5) $$w_{i,i+1}^{R}=-\int_{i}^{i+1}vdP$$

Once the dependence between *v* and *P* in the compression process is known, the integral can be numerically solved. This will be assessed in Section 3.1.

### 2.3. Compressor Efficiency and Specific Work

The characteristic thermodynamic parameter of a compressor is its isentropic efficiency, η, which compares the specific work that is required for a reversible compression against the specific work consumed by the real compression for an equal pressure ratio:

(6) $$\eta =\frac{w^{R}}{w}$$

where *w* indicates the real specific work, and *R* indicates the reversible case.

### 2.4. State Trajectory of a Compression

In general, the compression processes follow polytropic trajectories in the state space:

(7) $$Pv^{k}=C=P_{1}v_{1}^{k}$$

where *k* is the polytropic constant, usually between 1.2 and 1.3. For an isentropic process (adiabatic and reversible), k=1.41. In order to calculate a numeric value for $C=Pv^{k}$, the pressure and specific volume of the initial state can be substituted.

From the previous Equation (7):

(8) $$v={\left(\frac{P_{1}v_{1}^{k}}{P}\right)}^{1/k}={\left(\frac{C}{P}\right)}^{1/k}$$

## 3. Problem Solution

### 3.1. Specific Compression Work

Firstly, the specific compression work for a given compression stage can be formulated parting from (5) and (8):

(9) $$w_{i,i+1}^{R}=-\int_{i}^{i+1}vdP=-RT_{1}\frac{k}{k-1}(r_{i,i+1}^{\frac{k-1}{k}}-1)$$

In this expression, the basic hypotheses of the problem have been assumed: first, the cooling between consecutive compression stages bring the gas back to $T_{1}$ each time, so that the gas is always at this temperature at the start of any compression stage. Second, that the gas is an ideal gas. If the ideal gas hypotehsis is not assumed, compressibility factors at initial and final stages would appear [20].

The total specific work will be, according to (3):

(10) $$w^{R}=\sum_{i=1}^{n}w_{i,i+1}^{R}=-RT_{1}\frac{k}{k-1}\sum_{i=1}^{n}(r_{i,i+1}^{\frac{k-1}{k}}-1)$$

### 3.2. Optimisation

The problem consists in minimising (10) with the restriction given by (2). If this restriction were not considered, the obvious solution would result: $r_{i,i+1}=1,\;\forall i$, all compression ratios would be equal, and equal to 1; in other words, minimum work would occur when no compression took place. The Lagrange multipliers method requires minimising a Lagrangian function, *F*, instead of (10) directly, which integrates the restrictions that apply. A good summary of the method can be found in [14]. The *F* function to optimise would be:

(11) $$F=w^{R}-\lambda (\prod_{i=1}^{n}r_{i,i+1}-r_{t})$$

where λ is a parameter whose numerical value is calculated by imposing (2). *F* must be derived with respect to all variables, $r_{j,j+1},\;j=1\ldots n$. For greater clarity, the terms of the second member are independently derived for any given $r_{j,j+1}$:

(12) $$\begin{array}{ccc} \frac{\partial w^{R}}{\partial r_{j,j+1}} & = & -RT_{1}r_{j,j+1}^{-1/k} \end{array}$$

(13) $$\begin{array}{ccc} \frac{\partial \lambda \prod_{i=1}^{n}r_{i,i+1}}{\partial r_{j,j+1}} & = & \lambda \frac{\prod_{i=1}^{n}r_{i,i+1}}{r_{j,j+1}}=\lambda \frac{r_{t}}{r_{j,j+1}} \end{array}$$

Thus, with the condition of optimum ∀j:

(14) $$\frac{\partial F}{\partial r_{j,j+1}}=0=-RT_{1}r_{j,j+1}^{-1/k}-\lambda \frac{r_{t}}{r_{j,j+1}}$$

It must be taken into account that (14) represents *n* equations, for j=1…n. Additionally, yet, there is a single parameter λ common to all. Accordingly, the only way for this to hold is that all compression ratios be equal, the same value for all stages $r=r_{j,j+1}\;\forall j$. Returning to the condition (2), the value of *r* that optimises work can be deduced:

(15) $$\prod_{i=1}^{n}r=r^{n}=r_{t}\Rightarrow \begin{array}{c} r=r_{t}^{1/n} \end{array}$$

Hence, finally, the optimum specific compresion work is obtained by substitution in (10):

(16) $$w^{R}=-RT_{1}\frac{k}{k-1}n(r^{\frac{k-1}{k}}-1)$$

In the case that all compressors (stages) had the same efficiency η:

(17) $$\begin{array}{c} w=-\frac{1}{\eta }RT_{1}\frac{k}{k-1}n(r^{\frac{k-1}{k}}-1) \end{array}$$

The case in which each compressor had a different efficiency is less straightforward to formulate and interpret, and it will be assessed in Section 4.

### 3.3. Dimensioning of the Coolers

At the start of any given compression stage, between pressures i,i+1, for instance, the gas is at $T_{1}$. At the outlet it will be at $T_{i+1}$, which will depend on the compression ratio and the polytropic constant *k*.

The specific heat that will need to be extracted by the cooling will be the difference between the enthalpy of the gas at $T_{i+1}$ and that at $T_{1}$:

(18) $$q_{i,i+1}=c_{p}\left(T_{i+1}-T_{1}\right)$$

In case of adiabatic compression, which is: k=1.41, the heat would coincide exactly with the specific compression work. If not, it is necessary to calculate it by the temperatures. Temperature i+1, in the case of a reversible compressor, would be, according to Equation (8) and the ideal gas equation:

(19) $$T_{i+1}=T_{1}{\left(\frac{P_{i+1}}{P_{i}}\right)}^{\frac{k-1}{k}}=T_{1}r^{\frac{k-1}{k}}$$

Thus, substituting in (18) the specific heat results:

(20) $$\begin{array}{c} q_{i,i+1}=c_{p}T_{1}(r^{\frac{k-1}{k}}-1) \end{array}$$

It can be observed that it is identical for all stages, so the total heat to be extracted for the whole plant will be *n* times this.

## 4. Different Stage Efficiencies

The isentropic efficiency of the *n* stages of a compression might not be equal. Apart from the evolution of the thermodynamic properties of the gas from stage to stage, engineering requirements at each stage may impose limitations, so that the maximum theoretical efficiency cannot be reached; for example, in [15], designing a multistage compression of CO${}_{2}$ with centrifugal compressors, the maximum stress of the impeller is identified as the limiting condition for the impeller tip speed.

It is interesting to analyse this case, in order to analyse how the optimal compression ratio (15) should be varied at each stage to compensate for the differences in isentropic efficiency within the limits of the system (Section 4.1) and how the overall work is affected by having different stage isentropic efficiencies (Section 4.2).

### 4.1. Optimisation: Optimum Compression Ratio

The previous optimisation procedure can be generalised for different stage isentropic efficiencies. For an equal isentropic efficiency at all stages, minimising the reversible specific work $w^{R}$ of Equation (10) is equivalent to minimising the real work *w*, and so Equation (11) holds. However, the proportion in which inefficiencies at each stage will contribute to the total work will not be equal if the efficiencies differ, so the optimisation function F must be built with *w*, not $w^{R}$:

(21) $$\begin{array}{c} F=w-\lambda (\prod_{i=1}^{n}r_{i,i+1}-r_{t}) \end{array}$$

(22) $$\begin{array}{c} w=\sum_{i=1}^{n}w_{i,i+1} \end{array}$$

The specific work at each stage will be $w_{i,i+1}=\frac{1}{\eta_{i,i+1}}w_{i,i+1}^{R}$, where $w_{i,i+1}^{R}$ is the reversible specific work for the stage that is indicated in (9), and $\eta_{i,i+1}$ is the corresponding isentropic efficiency.

Now, analogously to (12) and (13):

(23) $$\begin{array}{ccc} \frac{\partial w}{\partial r_{j,j+1}} & = & -RT_{1}\frac{1}{\eta_{j,j+1}}r_{j,j+1}^{-1/k} \end{array}$$

(24) $$\begin{array}{ccc} \frac{\partial \lambda \prod_{i=1}^{n}r_{i,i+1}}{\partial r_{j,j+1}} & = & \lambda \frac{\prod_{i=1}^{n}r_{i,i+1}}{r_{j,j+1}}=\lambda \frac{r_{t}}{r_{j,j+1}} \end{array}$$

Thus, with the condition of optimum ∀i:

(25) $$\frac{\partial F}{\partial r_{j,j+1}}=0=-RT_{1}\frac{1}{\eta_{j,j+1}}r_{j,j+1}^{-1/k}-\lambda \frac{r_{t}}{r_{j,j+1}}\Rightarrow \lambda =-\frac{RT_{1}}{r_{t}}\frac{r_{j,j+1}^{\frac{k-1}{k}}}{\eta_{j,j+1}}$$

Again, (25) stands in reality for *n* equations, j=1,…,n, where there is a single parameter λ common to all. Hence, it must be:

(26) $$C=\frac{r_{j,j+1}^{\frac{k-1}{k}}}{\eta_{j,j+1}}$$

where *C* is a constant, whose value must satisfy the boundary condition (2). In order to force this, $r_{j,j+1}$ must be worked out previously:

(27) $$\begin{array}{cc} r_{j,j+1}=C^{\frac{k}{k-1}}\cdot \eta_{j,j+1}^{\frac{k}{k-1}} & \Rightarrow \end{array}$$

(28) $$\begin{array}{cc} r_{t} & =\prod_{j=1}^{n}r_{j,j+1}=C^{n\frac{k}{k-1}}\cdot \prod_{j=1}^{n}\eta_{j,j+1}^{\frac{k}{k-1}} \end{array}$$

From here, *C* can be worked out as a function of $r_{t}$ and $\eta_{j,j+1}$, and substituting back in (27), finally:

(29) $$r_{j,j+1}=r_{t}^{\frac{1}{n}}{\left(\frac{\eta_{j,j+1}}{\prod_{j=1}^{n}\eta_{j,j+1}^{\frac{1}{n}}}\right)}^{\frac{k}{k-1}}$$

It can be observed that, for the case of equal isentropic efficiency of all stages, $\eta_{j,j+1}=\eta \;\forall j$, this expression equals (15). On the other hand, (29) can be formulated in a more explicit way:

(30) $$r_{j,j+1}=r_{t}^{\frac{1}{n}}{\left(\frac{\eta_{j,j+1}}{\eta^{*}}\right)}^{\frac{k}{k-1}}=r_{0}{\left(\frac{\eta_{j,j+1}}{\eta^{*}}\right)}^{\frac{k}{k-1}}$$

The standard compression ratio for equal stage efficiencies given by (15) has been indicated by $r_{0}$. The denominator has been identified as the geometric mean of isentropic efficiencies: $\eta^{*}=\prod_{j=1}^{n}\eta_{j,j+1}^{\frac{1}{n}}$. The exponent in (15), as it can be deduced from (17), acts as a conversion factor between the pressure ratio and work.

Accordingly, it is interesting to observe how the standard pressure ratio value $r_{0}$ is scaled if the isentropic efficiency of a given compressor deviates from the geometric mean. A compressor whose efficiency coincided exactly with $\eta^{*}$ would be given the standard compression ratio: $r_{0}=r_{t}^{\frac{1}{n}}$. More efficient compressors would have to deliver higher ratios, the larger the greater their efficiency relative to $\eta^{*}$; the opposite would happen with less efficient compressors. With this strategy, the inefficiency that is generated by good compressors performing large pressure drops might tend to equal that of poor compressors doing small ones and, thus, the overall performance balanced within the possibilities of the system.

### 4.2. The Overall Effect of Different Efficiencies

The question arises as to how the performance of the system is affected by having different stage efficiencies. This can be analysed by formulating the total work (22):

(31) $$w=-RT_{1}\frac{k}{k-1}\sum_{i=1}^{n}\frac{1}{\eta_{i,i+1}}(r_{i,i+1}^{\frac{k-1}{k}}-1)$$

It is difficult to discuss over this expression, so it must be adapted. The weighted harmonic mean of the stage efficiencies, $\eta^{H}$ can be formulated:

(32) $$\eta^{H}=\frac{\sum_{i=1}^{n}(r_{i,i+1}^{\frac{k-1}{k}}-1)}{\sum_{i=1}^{n}\frac{1}{\eta_{i,i+1}}(r_{i,i+1}^{\frac{k-1}{k}}-1)}$$

where $\left(r_{i,i+1}^{\frac{k-1}{k}}-1\right)$ are used as weights for each $\eta_{i,i+1}$. Thus, with (31) and (32):

(33) $$w=-RT_{1}\frac{k}{k-1}\frac{1}{\eta^{H}}\sum_{i=1}^{n}(r_{i,i+1}^{\frac{k-1}{k}}-1)$$

The optimum pressure ratios of (30) have to be substituted, and also introducing the arithmetic mean of isentropic efficiencies: $\bar{\eta }=\frac{1}{n}\sum \eta_{i,i+1}$, after some operations:

(34) $$w=-\frac{1}{\eta^{H}}RT_{1}\frac{k}{k-1}n(r_{0}^{\frac{k-1}{k}}\frac{\bar{\eta }}{\eta^{*}}-1)$$

This expression is now similar to (17). It has to be noted that $\bar{\eta }\geq \eta^{*}\geq \eta^{H}$, the equality occurring only when efficiencies of all stages are equal, with this being a property of the pythagorean means [21]. Thus, the parenthesis will be greater than in (17). The fact that $\eta^{H}$ is more affected by lower values than higher values [22] must be remarked, even though the weighing of (32) could eventually alter this general trend.

Two interesting conclusions may be drawn. First, the tendency to increase compression work by poor compressors will tend to be stronger than the compensating tendency of efficient ones, although the weighing of (32) when using optimum compression ratios will contribute to equal them. Second, if a plant with different stage isentropic efficiencies were operated at equal compression ratios, $r_{0}$, instead of the optimum $r_{j,j+1}$ of (30), compression work would rise even higher.

For example, a four-stage compression with three 0.85 isentropic efficiency stages and one 0.79 stage would require 1.2% more optimum compression work than if all the stages had 0.85. If all stages were forced to operate at $r_{0}$ instead of the optimum ratios of (30), the compression work would be nearly 2% higher than with equal efficiencies, 0.7% higher than the work with optimum compression ratios.

## 5. Example with Reciprocating Compressors

Multistage compression stations increase the investment costs over single stage plants, so depending on mass flow and compression ratio requirements, and planned hours of operation, the latter, with higher operation costs, might result in being economically justified.

A brief example is going to be developed to illustrate the relation between compression ratio and design requirements in single and multistage plant design. The basics of reciprocating compressor operation are summarised in Section 5.1. A specific compressor will be analysed for a single stage compression in Section 5.2. The compressor will turn out not to be suitable. A multistage alternative using the same compressor will be analysed in Section 5.3.

### 5.1. Operating Parameters of a Compressor

The aspirated volume of gas on one cycle depends on the compression ratio as well as on the volume at the end of the compression, the clearance volume. It can be assumed that the ratio between the dead volume and that at the start is a=0.05. Somewhat higher values can also exist [20].

**Figure 2:**
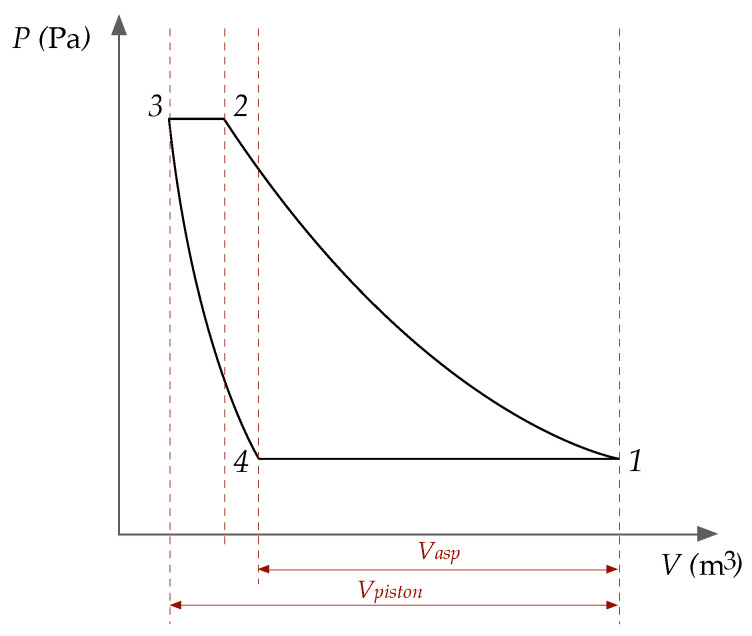
Complete cycle of a reciprocating compressor. The dead volume is $V_{3}$. Process 1-2: compression. Process 2-3: discharge. Process 3-4: expansion. Process 4-1: aspiration.

A compression cycle is schematically represented in Figure 2. Process 1-2 starts with the cyclinder full of gas at $\left(T_{1},P_{1}\right)$ conditions, which is compressed following the polytropic process described by (8). When the required pressure is reached, $P_{2}$, the discharge valve is opened and the gas is evacuated until the piston reaches the end of the stroke. At this point, the volume occupied by the gas is $V_{3}=a\cdot V_{1}$, the dead volume. This gas is approximately at $P_{2},T_{2}$. With closed admission and discharge valves, expansion takes place along another polytropic line until approximately $P_{1}$ in state 4. In process 4-1, gas enters from the exterior until the stroke is completed at $V_{1}$.

In the geometry of the Figure, it can be observed that, the higher the compression ratio, the closer will $V_{2}$ and $V_{3}$, so the amount of gas expelled in 3-2 will be smaller. If the compression ratio were raised enough, a point will be reached at which $V_{2}=V_{3}$, and no gas will be expelled when the discharge valve is opened, so the expansion line 3-4 will coincide with 1-2, and, therefore, no gas will be aspirated in 4-1.

This phenomenon is quantified by the *volumetric efficiency* of the compression process:

(35) $$\lambda =\frac{V_{asp}}{V_{piston}}$$

This parameter can be formulated in terms of *a*, and realising from (8) that $V_{4}=V_{3}\cdot r^{1/k}$:

(36) $$\lambda =\frac{1-a\cdot r^{1/k}}{1-a}$$

Therefore, the volume of gas that is really aspirated in a cycle will result: $V_{asp}=\lambda \cdot V_{piston}$. The piston will cover a volume given by bore and stroke, data supplied by the manufacturer.

### 5.2. Example 1: Dimensioning of a Compression Stage

A one-stage compression is required to process $50\;m^{3}/h$ of methane ($CH_{4}$) from 300 K, 1 bar to 18 bar. It must be established whether the following compressor can be used, given k≈1.2:
**no. cyl.**$N_{cyl}$2, single actingϕ × **stroke**
120 × 83 mm$P_{max}$18 bar$\dot{W}$22 kW*n*890 rpm*a*0.05

#### 5.2.1. Required Power

The minimum work required for one mole of gas, according to (9) would be:

(37) $$w^{R}=-RT_{1}\frac{k}{k-1}(r_{i,i+1}^{\frac{k-1}{k}}-1)=-9261.52\;J/\mathrm{mol}$$

By the ideal gas equation, 1 m${}^{3}$ of gas at inlet conditions contains 40.09 mol, so $w^{R}=-371294.34$ J/m${}^{3}$. The required volumetric flow is $50\;m^{3}/h=0.01389\;m^{3}/s$, making the required power ${\dot{w}}^{R}\approx 5.2$ kW. The compressor efficiency can be assumed η=0.9 [20], so the required power would result:

(38) $$\dot{w}\approx 5.8\;\mathrm{kW}<22\;\mathrm{kW}$$

The compressor would be valid.

#### 5.2.2. Flow Supply

The gas flow supplied by the compressor is proportional to the volume of gas aspirated in each cycle, $V_{asp}$. As it can be observed on Figure 2 (Section 5.1), it is a function of the geometric characteristics of the compressor and the compression ratio, r=18 in this case, given by the volumetric efficiency, λ.

Thus, the volume flow (${\dot{V}}_{CH_{4}}$) supplied by the compressor will be:

(39) $${\dot{V}}_{CH_{4}}=N_{cyl}\cdot n\cdot V_{piston}\cdot \lambda$$

The volumetric efficiency for this case, substituting the parameters of table (36), results in λ=0.467. That is, the compressor actually aspirates less than half the volume displaced by the piston on each cycle. Substituting in (39):

(40) $${\dot{V}}_{CH_{4}}=0.7803\;m^{3}/\min=46.82\;m^{3}/h<50\;m^{3}/h$$

Which shows that the compressor would not be valid because it does not reach the volume flow requirement. A different solution should be sought: compressors in parallel or multistage compression could be alternatives with reciprocating compressors, although other compressor technologies could perform better for large flows [18] and might be considered at this point.

#### 5.2.3. Maximum Compression Ratio for the Given Volume Flow

The maximum pressure at which the compressor could deliver the required $50\;m^{3}/h$ by substituting this value into (39) and working out λ and *r*:

(41) $$\lambda =\frac{{\dot{V}}_{CH_{4}}}{N_{cyl}\cdot n\cdot V_{piston}}=0.4987\Rightarrow r_{max}=16.85$$

### 5.3. Example 2: Multi-Stage Dimensioning

A three-stage compression facility must be designed for a $50\;m^{3}/h$ volume flow of methane ($CH_{4}$) from 300 K, one-bar to 18-bar using the compressor of Section 5.2, knowing k≈1.2.

#### 5.3.1. Optimum Case

By Equations (15) and (17) the optimum compression ratio, the specific compression works of each stage and the total are known:

(42) $$\begin{array}{c} r={\left(\frac{18\;\mathrm{bar}}{1\;\mathrm{bar}}\right)}^{1/3}=2.62 \end{array}$$

(43) $$\begin{array}{c} w_{i,i+1}=-20476.3\;J/\mathrm{mol} \end{array}$$

(44) $$\begin{array}{c} 50\;m^{3}/h=0.55681\;\mathrm{mol}/s;\;w=3\cdot w_{i,i+1}=-61428.95\;J/\mathrm{mol}\Rightarrow \end{array}$$

(45) $$\begin{array}{c} \Rightarrow \dot{W}=0.55681\;\mathrm{mol}/s\cdot -61428.95\;J/\mathrm{mol}=-34.2\;\mathrm{kW} \end{array}$$

On the other hand, according to (20) and $c_{p,CH4}=36.8$ J/molK, the cooling at the end of each stage results:

(46) $$q_{i,i+1}=-1922.4\;J/\mathrm{mol}\Rightarrow {\dot{Q}}_{i,i+1}=0.55681\;\mathrm{mol}/s\cdot q_{i,i+1}\approx 1.08\;\mathrm{kW}$$

accordingly, the total heat to be released is three times this: $\dot{Q}=3.24$ kW.

In these conditions, the volumetric efficiency is λ≈0.94, so, for the given r.p.m. and number of cylinders, the maximum volume flow the facility can deliver is $93.74\;m^{3}/h$.

#### 5.3.2. Off-Optimum Case

The case of setting intermediate pressures other than the optimal could be studied, in order to find out the difference in power requirement.

The case of splitting the total pressure drop in three equal stages can be considered. The total pressure drop is 17-bar, so three 5.6667 bar stages result (see the Table 1):

Applying η=0.9, and for the 0.5568 mol/s flow, the total compression power is $\dot{W}=-45.38$ kW, greater than the optimum −34.2 kW in (46), by nearly 33%. The total heat to be evacuated would increase from 3.24 to 3.34 kW.

## 6. Conclusions

The known formula for the optimal stage pressure ratio in a multistage compression, which assumes equal isentropic efficiency for all stages, has been deduced combining the Lagrange multiplier method and basic thermodynamics.

The same method has been used to analyse the case in which the isentropic efficiencies of each stage were not equal, reaching a formula for calculating the optimum compression ratio for each stage. The result shows that more efficient stages should be given a higher load than less efficient ones, in relation to how their isentropic efficiency deviates from the geometric mean of all isentropic efficiencies of the process. This novel result is interesting, as it is frequently the case that the isentropic efficiency of stages cannot be equal due to engineering limitations. It has been shown that poor compressors tend to influence the performance more than the efficient ones, and it has been established how operating all stages at an equal pressure ratio would increase the compression work even more.

An example of designing a multistage compression process with a reciprocating compressor has been developed to illustrate how component specifications relate to the optimum pressure ratio, the problems that might arise, and how they might be solved.

## Figures and Tables

**Table 1:** Pressure levels, specific work, and heat release in the off-optimum case.

Stage	bar	bar	–	J/mol	J/mol
	$P_{i}$	$P_{i+1}$	$r_{i,i+1}$	$w_{i,i+1}^{R}$	$q_{i,i+1}$
1-2	1.00	6.67	6.6667	−57,788.33	−4106.7
2-3	6.67	12.33	1.8500	−10,023.3	−1191.6
3-4	12.33	18.00	1.4595	−5532.9	−705.3
Total	1.00	18.00	18.0006	−73,344.53	−6003.8

## Abbreviations

The following abbreviations are used in this manuscript:

$e_{k}$	specific kinetic energy
$e_{p}$	specific potential energy
*F*	Lagrangian function
η	isentropic efficiency
$\eta^{*}$	geometric mean of all isentropic efficiencies
*i*	pressure level/number of stage
*k*	polytropic constant
λ	lagrange multiplier/volumetric efficiency
*n*	total number of compression stages/r.p.m. revolutions per minute
*P*	pressure
ψ	specific dissipation
*q*	specific heat
*r*	compression ratio
$r_{t}$	total compression ratio
*R*	ideal gas constant
*T*	absolute temperature
*v*	specific volume
*w*	specific work
$w^{R}$	specific reversible work
*W*	total work
Reciprocating compressors:	
*a*	ratio between clearance and swept volumes
λ	volumetric efficiency
*n*	r.p.m. revolutions per minute
$N_{cyl}$	number of cylinders
$V_{asp}$	aspirated volume
$V_{piston}$	volume swept by piston
